# Multimodal MRI Longitudinal Assessment of White and Gray Matter in Different SPG Types of Hereditary Spastic Paraparesis

**DOI:** 10.3389/fnins.2020.00325

**Published:** 2020-06-04

**Authors:** Domenico Montanaro, M. Vavla, F. Frijia, G. Aghakhanyan, A. Baratto, A. Coi, C. Stefan, G. Girardi, G. Paparella, S. De Cori, P. Totaro, F. Lombardo, G. Piccoli, Andrea Martinuzzi

**Affiliations:** ^1^U.O.C. Risonanza Magnetica Specialistica e Neuroradiologia, Fondazione CNR/Regione Toscana G. Monasterio, Pisa, Italy; ^2^Severe Developmental Disabilities Unit, Scientific Institute, IRCCS Eugenio Medea, Conegliano, Italy; ^3^U.O.C Bioengineering and Clinical Technology, Fondazione CNR/Regione Toscana G. Monasterio, Pisa, Italy; ^4^Department of Translational Research on New Technologies in Medicine and Surgery, Regional Center of Nuclear Medicine, University of Pisa, Pisa, Italy; ^5^Department of Radiology S. Maria dei Battuti Hospital – Conegliano, ULSS2-Marca Trevigiana, Conegliano, Italy; ^6^Institute of Clinical Physiology, National Research Council, Pisa, Italy; ^7^Acquired Neuropsychological Disease Rehabilitation Unit, Scientific Institute, IRCCS Eugenio Medea, Pieve di Soligo, Italy

**Keywords:** HSP, hereditary spastic paraplegia, MRS – 1H nuclear magnetic resonance spectra, VBM, voxel-based morphometry, DTI (diffusion tensor imaging), longitudinal analyze

## Abstract

Hereditary spastic paraplegias (HSP) are a group of genetically and clinically heterogeneous neurologic disorders. Hereby we describe a relatively large group of patients (pts) affected by HSP studied at baseline (31 pts) and at follow-up (mean period 28.9 ± 8.4 months; 23 pts) with multimodal advanced MRI: high-resolution T1 images for voxel-based morphometry (VBM) analysis, magnetic resonance spectroscopy (MRS), and diffusion tensor imaging (DTI). An age-matched healthy control (HC) group underwent the same neuroimaging protocol in a time schedule matched with the HSP patients. At baseline, VBM showed gray matter (GM) reduction in HSP in the right pre-frontal cortex and bilaterally in the thalami. MRS at baseline depicted in HSP patients compared to the HC group reduction of NAA/Cr ratio in the right pre-frontal region, increase of Cho/Cr ratio in the right pre-central regions, and increase of mI/Cr ratio on the left pre-central area. At cross-sectional follow-up analysis and longitudinal evaluation, no VBM and MRS statistically significant results were obtained. Tract-based spatial statistics (TBSS) analysis showed widespread DTI brain white matter (WM) alterations in patients compared to HC at baseline, which are characterized by reduction of fractional anisotropy (FA) and increase of mean diffusivity (MD), axial diffusivity (AD), and radial diffusivity, as confirmed on cross-analysis of the follow-up dataset. A longitudinal analysis with TBSS in HSP patients did not show significant variations, while upon applying region-based analysis we found increased FA and decreased MD and AD in specific brain WM fiber complex during follow-up. The changes were not correlated with the clinical presentation (pure *vs* complicated HSP), motor function, and motility indexes or history of specific treatments (botulinum toxin). In conclusion, the cross-sectional analysis of the multiparametric MRI data in our HSP patients confirmed the non-prominent involvement of the cortex in the primary motor regions but rather of other more associative areas. On the contrary, DTI demonstrated a widespread involvement of the brain WM, including the primary motor regions, which was confirmed at follow-up. The longitudinal analysis revealed an apparent inversion of tendency when considering the expected evolution of a neurodegenerative process: we detected an increase of FA and a decrease of MD and AD. These time-related modifications may suggest a repair attempt by the residual central WM fibers, which requires confirmation with a larger group of patients and with a longer time interval.

## Introduction

Hereditary spastic paraplegias (HSP) are a group of genetically and clinically heterogeneous neurologic disorders, also known as Strümpell–Lorrain disease (Faber et al., 2017), the authors who first described the clinical (Strümpell, 1880) and the neuropathological traits (Nayrac et al., 1953) of this disease.

The hinge points of HSP are progressive lower extremity spasticity with weakness and degeneration of the long axonal fibers of the cortico-spinal tract (CST) and the posterior cords (Fink and Hedera, 1999).

HSP is an inherited disease and the presence of a family history or consanguinity addresses toward a hereditary condition. Its absence, however, once other possible etiologies have been ruled out, should not dissuade the clinician from pursuing the search for a possible diagnosis of HSP (Faber et al., 2017).

Neuropathological reports on HSP are rare as the lifespan of these patients tends not to be shortened and they rarely die in hospital (Bisharat-Kernizan et al., 2018). Consequently, the pathological features first described in detail in 1952 (Schwarz, 1952) and subsequently categorized in 1974 (Behan and Maia, 1974) remain substantially the same at the moment. The common findings are represented by bilateral degeneration of the crossed CSTs with involvement of the uncrossed tracts, loss of Betz cells and spinal motor neurons, symmetrical degeneration of the gracile and cuneate fasciculi, mostly visible at thoracic level and increasing toward cervical regions up to the bulbar nuclei, and bilateral loss of fibers in the spinocerebellar tracts. No macroscopic lipid degeneration products and only occasional atrophy of the basal ganglia are reported (Erichsen et al., 2009). These postmortem studies suggest the degeneration of the distal ends of the longest motor and sensory axons as a possible pathogenetic mechanism. It is possibly due to a generalized failure of cells to maintain the vitality of terminal axons of more than a certain length, rather than a specific impairment of a particular neuronal system as observed in other neurodegenerative conditions, such as amyotrophic lateral sclerosis (ALS). Pathology descriptions suggest that the degeneration begins at the distal end of the axons and progresses toward the cell body, strengthening the hypothesis of a *dying back* axonopathy (Deluca et al., 2004). However, recent clinical and paraclinical studies revealed a wide involvement of the brain structures in HSP, including cerebellum, basal ganglia, and white matter (WM) of multiple brain regions, therefore suggesting how the simple *dying back* pathology is an incomplete unifying model (Aghakhanyan et al., 2014).

Classically, HSPs are subdivided on the basis of phenotypes into pure forms (pHSP), referring to the dominance of pyramidal signs, and complicated forms (cHSP), in which pyramidal findings are associated with dysfunction depending on other neurological systems, such as ataxia, parkinsonism, and cognitive impairment, or with systemic involvement, such as cataract, ichthyosis, etc. (Harding, 1983; Fink and Hedera, 1999). However, pure forms not rarely can show deficits typically seen in complicated forms, such as cognitive impairment (Jacinto-Scudeiro et al., 2019), and others seen in acquired motor neuron degeneration, like ALS (Denora et al., 2016), portraying the picture of a mosaicism of HSP phenotypes.

HSP have perhaps the inheritance patterns with the most striking heterogeneity among the neurogenetic diseases. The large genetic HSP heterogeneity is represented by over 85 spastic gait (SPG) disease loci and 79 known causative SPG identified genes (Parodi et al., 2018a; Coarelli et al., 2019). SPG4 type is the most frequent autosomal dominant subtype (Burguez et al., 2017). Genetic investigations have contributed to link HSP-related genes to other disorders, such as ALS and Alzheimer’s and Parkinson’s diseases (Novarino et al., 2014; Denora et al., 2016), or with genes previously associated with early-onset Charcot–Marie–Tooth disease and complicated encephalopathy (Parodi et al., 2018a; Olivieri et al., 2019). The identification of a number of casual genes and the determination of their products’ dysfunction is helping to better understand common elements underlying the cellular process involved in the disease (Boutry et al., 2019).

Diagnosing HSP is a difficult task due to the high degree of genetic heterogeneity and the wide spectrum of clinical features that may confound HSP with other neurological diseases, particularly in patients without a positive family history (Cui et al., 2018). In this widely heterogeneous clinical and genetic landscape, magnetic resonance imaging (MRI) of the brain is fundamental as it allows to exclude other diseases such as acquired myelopathies, primary progressive multiple sclerosis, vitamin B12 deficiency, copper deficiency, spinal cord tumors or malformations, and degenerative disorders of the spine. Conventional MRI of the brain is usually normal in pHSP, and non-specific findings are present in cHSP (cortical atrophy and subcortical and periventricular WM alterations; Kassubek et al., 2006). Thin corpus callosum (TCC), brain iron accumulation, and cerebellar hypoplasia/atrophy are findings associated in HSP with intellectual disability or cognitive involvements. TCC is frequent in SPG11 and SPG15, even if it can be occasionally present in other forms, collectively labeled as “HSPs with TCC subgroup” (Abdel Aleem, 2017).

In the last decades, among all the neuroimaging field, the so-called advanced MRI techniques have been widely applied as useful tools in HSP. These include voxel-based morphometry (VBM) and diffusion tensor imaging (DTI) (Graça et al., 2019) that demonstrated *in vivo* structural abnormalities correlated with disease mechanisms and progression (Faber et al., 2018). Their specific added benefits consist in the possibility to perform automated and unbiased whole-brain analyses and to localize *in vivo* the distribution of neural damage not otherwise visually evaluable (França et al., 2012).

Magnetic resonance spectroscopy (MRS) is another MRI advanced technique that enables the biochemical characterization of disease-related cerebral abnormalities, identifying either specific metabolites or variations of the most frequently reported brain macromolecules (Dreha-Kulaczewski et al., 2006; Schneider-Gold et al., 2017), among which reduction of Choline (Cho) and N-Acetyl Aspartate (NAA) were described in SPG4 patients compared to age-matched healthy controls (HC) in frontal WM and motor cortex, with a trend of an increase of myo-inositol, indicating neuro/axonal dysfunction and astroglia-mediated repair attempt (Erichsen et al., 2009).

DTI is applied to characterize the *in vivo* microstructural organization of the WM. The common DTI indices are fractional anisotropy (FA), sensitive to microstructural changes and associated with the presence of oriented structures in tissues, and mean diffusivity (MD), which is proportional to the overall presence of obstacles to diffusion. Other indices, such as axial diffusivity (AD) and radial diffusivity (RD), are elements useful to differentiate axonal injury and demyelination (Aghakhanyan et al., 2014).

Longitudinal changes occurring over time in the brains of HSP patients are still unclear: for example, disease progression seems to differ between the various HSP forms because little or no progression is described in some (SPG3), while a clear worsening is documented in others (SPG4) even within a few years (Schneider-Gold et al., 2017). Defining the extent and the longitudinal progression of brain involvement is fundamental in neurodegenerative diseases for understanding the pathophysiology, assessing prognosis, and thus improving care and monitoring experimental treatments in therapeutic trials. In this context, MRI has provided several biomarker candidates that may be offered as probes for the investigation of the natural disease progression and the effectiveness of therapeutic approaches (Menke et al., 2018). In particular, several types of advanced MRI techniques have been proposed as objective measures of dysfunctions in various motor neuron diseases, such as in ALS (Senda et al., 2011; Keil et al., 2012; Kwan et al., 2012). The same methodologies were proposed for the study on HSP, aimed to cross-sectional observations, while available longitudinal studies are poor and mostly technically limited (de Albuquerque et al., 2017).

This study was designed to reproduce literature data on the role of a multimodal advanced MRI approach (DTI, VBM, and MRS) in defining disease-specific alterations in HSP. Following the same group of HSP patients and HC over time and with the identical MRI methodology, we aimed also to evaluate longitudinal brain changes in HSP, possibly identifying specific anatomic involvement and types of brain modifications, in one of the relatively larger group of HSP patients ever reported.

## Materials and Methods

### Patients

Thirty-one patients affected by HSP (age 43.5 ± 12 mean ± SD) attended at the Medea Scientific Institute in Conegliano (TV), Italy, and 36 HCs matched by age and gender (age 43.65 ± 10, mean ± SD) were recruited from 2009 to 2017. All the HSP patients underwent molecular genetic studies. Disease severity measures were assessed with various clinical tests, including the Spastic Paraplegia Rating Scale (SPRS) (Schüle et al., 2006) that was the only clinical measure used for correlation with the MRI variables.

### Imaging Protocol

All the subjects were included in the protocol on the basis of the common criteria for a MRI study. They were examined with a 1.5 Tesla equipment (Philips Achieva 2.5 XR, Royal Philips Healthcare, Eindhoven, NL) at baseline (*T*_0_) and at follow-up (*T*_1_).

Depending on the quality of the single acquisitions in the presence of movement artifacts, datasets were discarded. From the initial group of subjects, 23 patients and a corresponding 23 age-matched HC group, after the basal MRI examination (*T*_0_), underwent MRI follow-up (*T*_1_) adopting the same standard and advanced sequences. The mean interval between the two examinations was 28.9 ± 8.4 months, corresponding to 2.40 ± 0.70 years. MRI data were analyzed cross-sectionally (at *T*_0_ and at *T*_1_) and longitudinally (*T*_0_ *vs T*_1_).

The study was approved by the Institutional Ethics Committee of the IRCCS “Eugenio Medea” Research Institute (# 63/09CE) and was conducted in accordance to the ethical standards of the Declaration of Helsinki (1964). All the adult participants and parents or legal tutors provided written informed consent prior to inclusion in the study. All the related documents were collected and stored by the clinical investigators of the center (MV and AM) according to the Institutional Ethic Committee guidance.

The MRI examinations included a standard protocol in order to exclude other pathologies (T2, proton density, and T1-weighted images). Sequences dedicated to the present study were obtained as follows:

T1-weighted 3D isotropic images with 1 mm voxel size (turbo-FFE, TR/TE 7.1/3.2, acquisition matrix 260 × 232, turbo FE factor 232, NSA 1);

Single-voxel H1-MR spectroscopy (MRS) localized in WM/gray matter (GM) of the bilateral pre-central (for primary motor functions) and in pre-frontal regions (for associative motor functions), for a total of four samplings for each study (PRESS sequence: TR 2000 ms, TE 35 ms, voxel size 15 × 15 × 15 mm);

DTI acquisition with single-shot FLAIR EPI-Echo Planar Image on axial plane (TR 10000 ms, TE 69 ms; IR 2400 ms; EPI factor 55; acquisition matrix 104 × 102; *b*-value 0–1000 s/mm^2^; 102 contiguous slices), voxel size 2 × 2 × 2 mm; 32 different gradient directions.

### Voxel-Based Morphometry

VBM evaluations were performed with FSL-VBM (Douaud et al., 2009), an optimized VBM protocol (Good et al., 2001) carried out with FSL tools (Smith et al., 2004). First, the structural images were brain-extracted and cortical and deep GM was segmented before being registered to the 2-mm MNI 152 standard space using non-linear registration (Andersson et al., 2007a, b). The resulting images were averaged and flipped along the x-axis to create a left–right symmetric, study-specific GM template. Then, all the native GM images were non-linearly registered to this study-specific template and “modulated” to correct for local expansion (or contraction) due to the non-linear component of the spatial transformation. During the modulation step, each voxel of every registered GM image was multiplied by the Jacobian of the warp field (Good et al., 2001). This defines the direction (larger or smaller) and the amount of modulation. The modulated GM images were then smoothened with an isotropic Gaussian kernel with a sigma of 3 mm.

### Magnetic Resonance Spectroscopy

The major brain metabolites detectable with H-MRS were analyzed: N-acetyl aspartate (NAA, processing at 2 ppm, marker for axonal density), choline (Cho, 3.2 ppm, index of membrane turnover), creatine-phosphocreatine complex (Cr, index of cell/axonal energy metabolism), and myo-inositol (mI, 3.5 ppm, index of glial activity). To define a peaks’ table for each metabolite, data were processed with *Spectro View Software* (Philips Healthcare, NL), measuring peaks and areas under the curves as ratio referred to the Cr, which was considered as normative inter-subject units (Han and Ma, 2010).

### Diffusion Tensor Imaging

Whole-brain DTI analyses were performed using tract-based spatial statistics (TBSS) in FSL version 4.1.1 (Smith et al., 2004), the Oxford Centre for Functional MRI of the Brain (FMRIB)^1^ software library. First, raw diffusion data were corrected for motion artifacts and eddy current distortions with an automated procedure; then, the brain was extracted using the brain extraction tool (Smith, 2002). The FMRIB’s diffusion toolbox (Behrens et al., 2003) was used to fit the diffusion tensor and compute the diagonal elements (eigenvalues λ1, λ2, and λ3) for each brain voxel, from which the derived metrics FA, MD, AD, and RD matrix were calculated for each subject. Voxelwise statistical analysis of these matrices was carried out with TBSS (Smith et al., 2006), part of FSL. All subjects’ FA data aligned into a common space (1 × 1 × 1 mm FMRIB58_FA standard space was used as target images), applying the non-linear registration tool FNIRT (Andersson et al., 2007a, b). Next, the mean FA image was created and thinned (threshold FA value of 0.3) to create a mean FA skeleton which represents the center of all tracts common to the group. Finally, all subjects’ spatially normalized FA, AD, RD, and MD data were projected onto the skeleton and the resulting data were analyzed into voxelwise cross-subject statistics. Individual skeleton images were submitted to a general linear model (GLM) analysis with appropriate design matrices and linear contrasts defined for the group comparisons.

A DTI region-based analysis (ROI) was applied using atlas-based predefined brain regions on the basis of the JHU White Matter Tractography and Harvard–Oxford Cortical Structural Atlas (part of FSL) (Mori et al., 2005). The ROIs were projected on the FA, MD, RD, and AD maps, and the mean values of each of the DTI indices were extracted using fsl meant tool (part of FSL).

### Statistical Analysis

To assess gray and white matter decline at baseline and over the time, different comparisons between groups were performed: (1) comparison between HSP patients and HC group, (2) difference between pure and complicated form, (3) comparison between the SPG4 patients and the HC group, (4) SPG4 compared with other SPG forms, and (5) analysis of longitudinal changes from baseline and follow-up. Correlation with SPRS scale and treatment with botulinum toxin were tested within the HSP group.

For the VBM analysis, a design matrix for a GLM was constructed in FSL to compare the GM differences between two groups accounting for age as a nuisance covariate. Threshold-free cluster enhancement (TFCE)-based analysis was applied with 5000 permutations (Bullmore et al., 1999; Hayasaka and Nichols, 2004), which is an optimized method to detect clusters without having to define clusters in a binary way (Smith and Nichols, 2009). Family-wise error rate (FWE) was controlled and FWE-corrected *p*-values less than 0.05 were accepted.

The MRS data were analyzed in each investigated area (pre-central and pre-frontal, right and left) with Student’s *t*-test or the non-parametric unpaired Wilcoxon test for continuous variables, evaluating differences in concentration of metabolite mean values with a confidence interval of 95% (CI95). In all analyses, a two-sided *p* < 0.05 was considered as statistically significant. Statistical analyses were performed using STATA software (StataCorp. Stata Statistical Software: Release 13, StataCorp LP, College Station, TX, United States, 2013).

DTI-based voxelwise statistics were carried out using the program Randomize, part of FSL, which uses Monte Carlo permutation testing to generate *n* number of random permutations for non-parametric statistics (Nichols and Holmes, 2002). With this approach, voxelwise differences among groups applying two-sample *t*-tests were assessed. We used the thresholded mean FA skeleton (mean value of 0.3), setting the number of permutations to 5000 with TFCE option and the significance threshold at *p* = 0.05 corrected for multiple comparisons to control FWE. The data were analyzed accounting for age as a nuisance covariate.

In order to implement a longitudinal design matrix for statistical analysis for VBM and TBSS, we subtracted each time-point 0 image from the time-point 1 image. All subsequent whole-brain statistical analyses (the same applied at the baseline study) were performed on these different images.

The GLM randomized test was applied also using SPRS indices as a covariate for correlations between imaging measures (VBM and DTI maps).

## Results

All the HSP patients were molecularly defined with confirmed mutations in the SPG loci, except for one patient who, at the time of this study, had not yet been molecularly characterized but whose clinical history and presentation were strongly suggestive of a pHSP; so he was included in the list, as other authors suggest (Faber et al., 2017). The SPG types of all HSP patients are reported in Table 1. Their distribution reflects that described in most studies conducted on Western Europe population (Bisharat-Kernizan et al., 2018).

**TABLE 1:** Demographic and clinical data of HSP patients and healthy control group.

	HSP patients	Healthy controls
	*N* = 31	*N* = 36
Gender	16 F, 15 M	26 F, 10 M
Disease onset (years)	27 ± 17	
Age at first MRI (years)	43.5 ± 12	43.65 ± 10
Age at follow-up MRI (years)	44 ± 12.8	47.8 ± 7.4
Disease duration at first MRI (years)	14 ± 13	
Disease duration at follow up MRI (years)	18 ± 13	
**SPastic Gate gene**		
SPG4	40% (12)	
SPG5	13% (4)	
SPG30	10% (3)	
SPG3	10% (3)	
SPG8	6% (2)	
SPG11	6% (2)	
SPG7	3% (1)	
SPG72	3% (1)	
SPG10	3% (1)	
SPG31	3% (1)	
SPG not defined	3% (1)	

On the basis of the clinical presentation, HSP patients were subdivided into pure (16 patients, 52%) or complicated forms (15 patients, 48%).

The phenotypical characteristics of the patients were represented by lower limbs (LL) clonus (seven patients, 23%), LL dyskinesias (one patient, 3%), muscle hypo-atrophy (five patients, 16%), hypo-pallesthesia (12 patients, 39%), cerebellar involvement (seven patients, 23%), dystonic tremor (one patient 3%), nystagmus (two patients, 6%), urinary urgency (two patients, 6%), and axonal neuropathy (one patient, 3%). The neuropsychological profiles were characterized by deficits in spared tests as follows: in five patients (16%) deficits of verbal fluency, in 11 patients (35%) deficits of memory, and in 10 patients (32%) deficits of attention, praxia, and visuo-spatial perception.

At visual inspection and qualitative assessment, MRI showed on standard morphological images normal aspects in almost a third of all tested patients. Alterations in deep supratentorial WM, diffuse or focal brain atrophy, and the “ear of the lynx” sign were detected in some cases, with percentage matched with our previous report (Martinuzzi et al., 2016): WM alterations in 32%, corpus callosum atrophy in 16%, “ears of lynx” sign in 19%, and visually evaluable cortical and/or cerebellar atrophy in 40%.

### Advanced MRI Results

#### At Baseline

**FIGURE 1:**
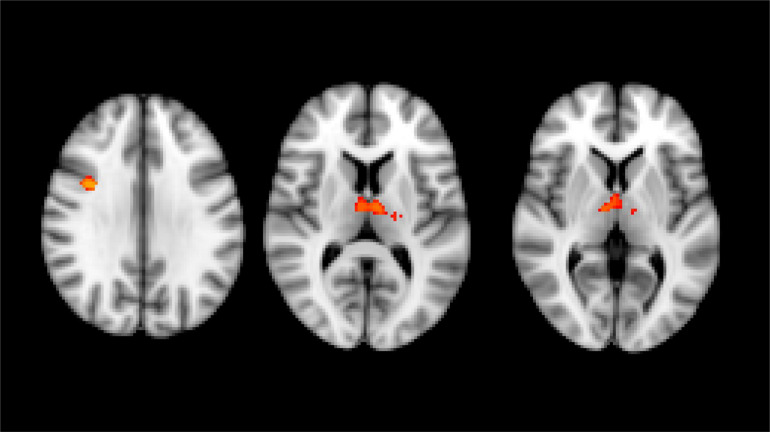
Results of whole brain voxel-based morphometry analyses superimposed on the T1-weighted structural MRI images in the standard MNI space. T-statistics of the clusters with significant changes in between-group VBM analysis at baseline. Red-yellow clusters show gray matter volumetric reduction in HSP patients compared with healthy control group (right prefrontal cortex and both thalami).

VBM analysis performed with whole-brain volumetric method at the baseline revealed a volumetric loss in HSP patients compared to the HC group (number of subjects in each group = 31) in the right pre-frontal cortex and bilaterally in both thalami (*p* < 0.05, FWE corrected) (Figure 1). No statistical correlation was found stratifying by clinical presentation (pure *vs* complicated forms), disease severity measure (SPRS), history of specific treatments (chemodenervation using botulinum toxin or antispasticity drugs), and disease duration.

Statistical differences were detected in MRS spectra (Table 2): HSP patients showed increased mI/Cr ratio in the left pre-central area, lower NAA/Cr in HSP than HC in the right pre-frontal area, and higher Cho/Cr in HSP than HC in the right pre-central region. Comparing SPG4 patients and HC, higher levels of NAA in the left pre-frontal of HC than of SPG4 were detected. Comparing the various SPG subtypes within HSP patients, mI/Cr in non-SPG4 types were significantly higher than in SPG4 patients in the left pre-central area and in the right pre-frontal region.

**TABLE 2:** Metabolite ratios on MRS samplings of HSP patients and control group at baseline.

	R pre-central			L pre-central			R pre-frontal			L pre-frontal		
	Patients	Control	*p*-value	Patients	Control	*p*-value	Patients	Control	*p*-value	Patients	Control	*p*-value
	(*n* = 31)	(*n* = 31)		(*n* = 31)	(*n* = 31)		(*n* = 16)	(*n* = 14)		(*n* = 16)	(*n* = 14)	
NAA/Cr	1.72 ± 0.21	1.74 ± 0.18	ns	1.75 ± 0.16	1.81 ± 0.15	ns	**1.70 ± 0.16**	**1.84 ± 0.20**	**0.040**	1.95 ± 0.29	2.09 ± 0.21	ns
Cho/Cr	**0.77 ± 0.13**	**0.70 ± 0.11**	**0.032**	0.75 ± 0.14	0.77 ± 0.12	ns	0.74 ± 0.11	0.75 ± 0.15	ns	0.73 ± 0.11	0.78 ± 0.13	ns
myo-Ins/Cr	0.61 ± 0.24	0.56 ± 0.08	ns	**0.64 ± 0.12**	**0.57 ± 0.09**	**0.018**	0.66 ± 0.14	0.61 ± 0.12	ns	0.64 ± 0.16	0.62 ± 0.08	ns

*NAA, N-acetyl-aspartate; Cr, creatine; Cho, choline; myo-Ins, myo-inositol; ns, not significant. Bold characters represent values with *p* < 0.05.*

**FIGURE 2:**
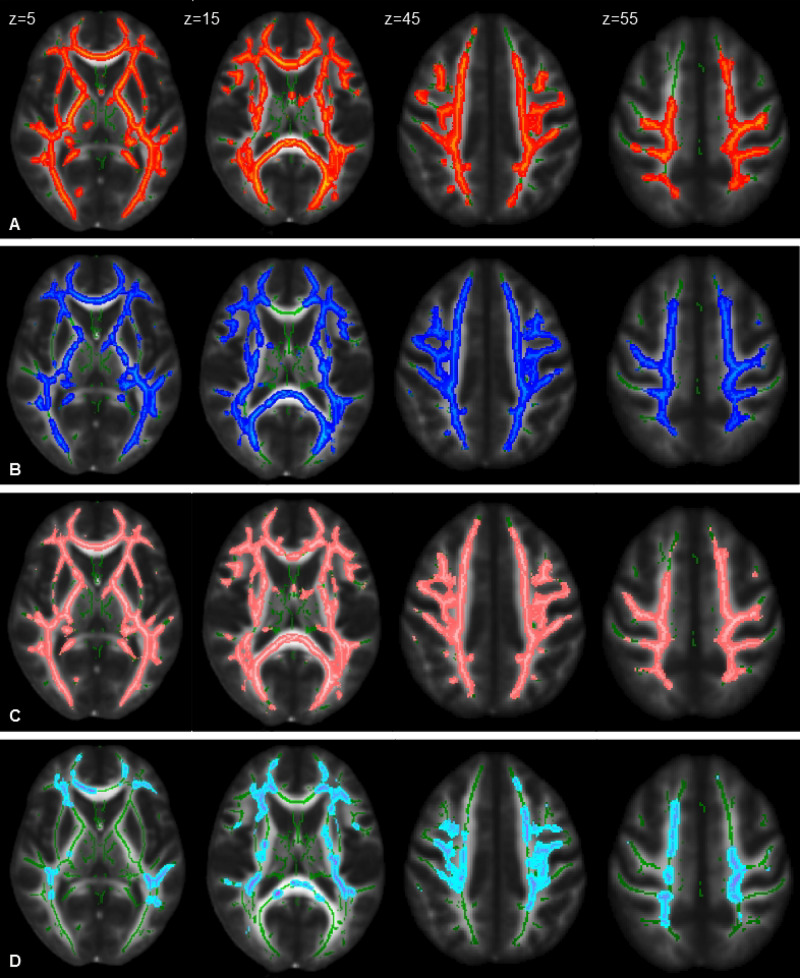
Statistical maps of DTI indexes (*P* < 0.05, FWE corrected) in HSP patients compared with healthy control group at the baseline represented on the FMRIB58_FA_1mm template. Images correspond to the same z-axis coordinates (in millimeters), reported on the left top of images on the first raw; green lines represent the mean white matter skeleton of all participants. Referred to HSP patients: **(A)** red-yellow, areas of decreased FA; **(B)** blue-light blue, areas of increased MD; **(C)** pink-light pink, areas of increased RD; **(D)** cold-light blue areas of increased AD.

An analysis of DTI data with TBSS revealed significant differences bilaterally in widespread brain regions (*p* < 0.05, FWE corrected, 28 HSPs – three were excluded for poor quality of images – and 36 HCs): CST, cingulum, corpus callosum, inferior fronto-occipital fasciculi, inferior longitudinal fasciculi, and superior longitudinal fasciculi. In particular, we observed patients with reduced FA (including parts of the brainstem) and increased MD and AD (not involving brainstem) and RD (involving the brainstem) (Figure 2). No area with increased FA and decreased MD was detected.

In a subgroup analysis comparing only SPG4 (10 subjects, two were excluded for technical reasons) and healthy subjects (36 subjects), the patients showed reduction of FA and increase of MD and RD in widespread regions; AD was also increased, but only on the right side of the corpus callosum, corona radiate, and semioval center. There were no differences between SPG4 (10 subjects) and the other SPG forms (18 subjects) in any of the DTI measurements. In HSP patients, no statistical correlation was found between DTI indices and SPRS and between patients with and without history of treatments with botulinum toxin.

#### Follow-up and Longitudinal Analysis

On cross-sectional analysis at follow-up and at the longitudinal analysis, VBM comparing residual 23 HC and 23 patients did not reveal any statistically significant variation. Similarly, MRS at follow-up cross-sectional analysis and at longitudinal comparisons did not show any significant change (41 samples for pre-central areas – 17 HC and 24 patients and 33 for pre-frontal region – 13 HC and 20 patients).

Comparing at follow-up the DTI results in HSP patients (valid for analysis 19 patients) with those in HCs (18 subjects), we confirmed the presence of significantly reduced FA values (with the sparing of the brainstem) and higher MD and RD (*p* < 0.05, FEW-corrected); no statistically significant differences were detected for AD.

The longitudinal analysis (*T*_0_ *vs T*_1_) by whole-based voxelwise paired TBSS did not detect for all the DTI indices any significant differences in the patient group (19 subjects). No statistical correlation was found in the HSP longitudinal data with SPRS and history of specific treatments (chemodenervation with botulinum toxin).

Region-based analysis in the same group of HSP patients identified statistically significant differences as reported in Table 3: at *T*_1_, the FA values increased (left superior longitudinal fasciculus *p* < 0.05, right pre-central gyrus *p* < 0.02, left pre-central gyrus *p* < 0.03, right post-central gyrus *p* < 0.04, left post-central gyrus *p* < 0.02); decreased values were detected for MD (left CST *p* < 0.03, forceps major *p* < 0.01, forceps minor *p* < 0.03, left superior longitudinal fasciculus *p* < 0.03, right pre-central gyrus *p* < 0.02, left pre-central gyrus *p* < 0.02, right post-central gyrus *p* < 0.04, left post-central gyrus *p* < 0.009) and RD (left CST *p* < 0.02, right cingulum *p* < 0.03, left cingulum *p* < 0.02, forceps major *p* < 0.02, forceps minor *p* < 0.03, left inferior fronto occipital *p* < 0.04, left superior longitudinal fasciculus *p* < 0.02, right pre-central gyrus *p* < 0.01, left pre-central gyrus *p* < 0.02, right post-central gyrus *p* < 0.03, left post-central gyrus *p* < 0.006); conversely, the AD values varied without a constant tendency (at *T*_1_ increased with respect to *T*_0_ in the left superior longitudinal fasciculus *p* < 0.05 and left post-central gyrus *p* < 0.02; at *T*_1_ decreased with respect to *T*_0_ in left *p* < 0.03 and right *p* < 0.02 pre-central gyri and right post-central gyrus *p* < 0.04).

**TABLE 3:** DTI mean values in HSP patients at baseline (*T*_0_) and at follow-up (*T*_1_) and statistical results in longitudinal comparison (*p*-values of differences *T*_0_ *vs T*_1_).

Fiber bundles	Groups	FA (mean ± SD)	MD (mean ± SD)*	RD (mean ± SD)*	AD (mean ± SD)*
L corticospinal tract	HSP *T*_0_	0.496 ± 0.028	0.687 ± 0.028	0.472 ± 0.035	1.117 ± 0.032
	HSP *T*_1_	0.508 ± 0.028	0.678 ± 0.030	0.460 ± 0.031	1.122 ± 0.036
	*p*-value	ns	**0.03**	**0.02**	ns
L cingulum	HSP *T*_0_	0.467 ± 0.036	0.719 ± 0.030	0.517 ± 0.039	1.122 ± 0.030
	HSP *T*_1_	0.481 ± 0.035	0.712 ± 0.046	0.504 ± 0.039	1.080 ± 0.104
	*p*-value	ns	ns	**0.02**	ns
R cingulum	HSP *T*_0_	0.407 ± 0.039	0.723 ± 0.026	0.552 ± 0.037	1.065 ± 0.032
	HSP *T*_1_	0.428 ± 0.052	0.718 ± 0.042	0.537 ± 0.037	1.128 ± 0.080
	*p*-value	ns	ns	**0.03**	ns
Corpus callosum with forceps major	HSP *T*_0_	0.496 ± 0.028	0.834 ± 0.046	0.575 ± 0.046	1.352 ± 0.061
	HSP *T*_1_	0.505 ± 0.027	0.822 ± 0.048	0.561 ± 0.045	1.343 ± 0.071
	*p-*value	ns	**0.01**	**0.02**	ns
Corpus callosum with forceps minor	HSP *T*_0_	0.413 ± 0.029	0.787 ± 0.054	0.595 ± 0.057	1.172 ± 0.052
	HSP *T*_1_	0.425 ± 0.023	0.775 ± 0.051	0.579 ± 0.047	1.168 ± 0.066
	*p*-value	ns	**0.03**	**0.03**	ns
L inferior fronto-occipital longitudinal fasciculus	HSP *T*_0_	0.424 ± 0.031	0.799 ± 0.043	0.599 ± 0.050	1.198 ± 0.038
	HSP *T*_1_	0.435 ± 0.023	0.792 ± 0.040	0.588 ± 0.042	1.191 ± 0.053
	*p*-value	ns	ns	**0.04**	ns
L superior longitudinal fasciculus	HSP *T*_0_	0.389 ± 0.023	0.738 ± 0.040	0.579 ± 0.041	1.056 ± 0.044
	HSP *T*_1_	0.397 ± 0.023	0.729 ± 0.042	0.558 ± 0.042	1.061 ± 0.058
	*p*-value	**0.05**	**0.03**	**0.02**	**0.05**
L pre-central gyrus	HSP *T*_0_	0.163 ± 0.013	0.488 ± 0.029	0.426 ± 0.030	0.612 ± 0.028
	HSP *T*_1_	0.176 ± 0.022	0.471 ± 0.012	0.406 ± 0.014	0.584 ± 0.022
	*p*-value	**0.03**	**0.02**	**0.02**	**0.03**
R pre-central gyrus	HSP *T*_0_	0.154 ± 0.010	0.476 ± 0.024	0.416 ± 0.025	0.595 ± 0.023
	HSP *T*_1_	0.167 ± 0.022	0.459 ± 0.012	0.396 ± 0.015	0.601 ± 0.023
	*p*-value	**0.02**	**0.02**	**0.01**	**0.02**
L post-central gyrus	HSP *T*_0_	0.133 ± 0.010	0.407 ± 0.026	0.355 ± 0.026	0.509 ± 0.027
	HSP *T*_1_	0.143 ± 0.015	0.391 ± 0.009	0.337 ± 0.011	0.510 ± 0.015
	*p*-value	**0.02**	**0.009**	**0.006**	**0.02**
R post-central gyrus	HSP *T*_0_	0.136 ± 0.008	0.413 ± 0.023	0.360 ± 0.023	0.519 ± 0.023
	HSP *T*_1_	0.145 ± 0.014	0.400 ± 0.010	0.345 ± 0.011	0.497 ± 0.015
	*p*-value	**0.04**	**0.04**	**0.03**	**0.04**

White matter tracts labeled according to probabilistic JHU White-Matter Tractography Atlas. L, left; R, right. * × 10^–3^ mm^2^/s. NS, not significant. Bold characters indicate values with *p* < 0.05.

A longitudinal clinical evaluation with SPRS in the entire cohort of our patients did not reveal any significant modification; also, upon evaluating SPG4 and non-SPG4, we did not depict variations.

The longitudinal evaluation of the HCs (18 subjects) did not show any modification in DTI indices, both applying the whole-based voxelwise paired TBSS analysis and the region-based analysis.

## Discussion

In this study, we explored cerebral changes in a relatively large group of molecularly characterized HSP patients by applying advanced MRI methods: voxel-based morphometry, to assess cortical and deep GM; MRS to sample correlated metabolic changes in pre-central and pre-frontal regions; and diffusion tensor imaging with multiple diffusivity indices to evaluate WM microstructural alterations. The most relevant data is a variation of DTI values at longitudinal analysis, suggestive of an improvement of the WM organization.

Our data obtained with both VBM and MRS revealed scattered brain modifications in HSP patients compared to HCs but did not show macroscopic primary motor cortex modifications to be possibly correlated to the neurodegenerative process underlying HSP.

VBM identified a reduction of the brain volume in the right pre-frontal cortex and bilaterally in the thalami. MRS revealed in patients decreased NAA/Cr in pre-frontal regions, an expression of neuronal/axonal pauperization or hypo-functionality, but not in the primary pre-central motor regions. The ratio mI/Cr, probably related to a complex gliotic and astrogliotic reaction, was increased in the pre-central regions. No variation was detected on the longitudinal analysis both for VBM and MRS possibly because of the reduced number of subjects (from 31 HSPs and 36 HCs to 23 HSPs and 23 HCs) and the poor quality of some MRS samplings. As an example, of a total of 184 MRS samplings expected for all the subjects at follow-up, only 53 samplings in pre-central regions and 42 in pre-frontal areas were acceptable for analysis.

The cross-sectional analysis of DTI data at baseline and at follow-up identified in HSP patients a widespread impoverishment of the WM microstructure, involving also the primary motor regions, similarly to the previous literature reports. Interestingly, in this study we observed a significant inversion of DTI trends on longitudinal analysis in HSP patients, with an increase in FA and decrease in MD in multiple cerebral WM regions. Such changes paradigmatically can be an expression of a structural improvement that is fascinating and intriguing, but complicated to discuss.

The major feature in HSPs is an axonal degeneration, which is maximal at the terminal portions of the longest descending and ascending tracts as confirmed by modern neuroimaging studies (Graça et al., 2019). The extension of the neurodegenerative process to the cerebral WM is mainly produced by a *dying back* degeneration (Deluca et al., 2004; Hourani et al., 2009). Biochemical studies, supported by clinical and neuroimaging observations, pointed out that *dying back* degeneration cannot be the only pathophysiologic mechanism as in HSP there is a widespread brain and spinal involvement that extends well beyond the primary motor and sensory areas (Erichsen et al., 2009; Aghakhanyan et al., 2014; Graça et al., 2019; List et al., 2019). The wide variability in the clinical presentation of patients with HSP itself and the variability in genetic background suggest that damage is not restricted to the CST in most SPG forms.

In this heterogeneity, MRI demonstrated to be an efficient neuroimaging technique in revealing meaningful changes in HSP. In our patients, standard MRI did not reveal many alterations and those that are considered indicative of HSP are very few, as reported in literature (Ortega and Rosemberg, 2019). Conversely, the so-called advanced MRI assesses the CNS quantitatively and proves to be an exquisite sensitivity tool for subtle anatomical abnormalities higher than the pure visual analyses.

Among the advanced MRI techniques, VBM aims to identify local differences of brain tissue by analyzing groups of 3D MRI datasets. It can include cortex and deep GM and can be assessed with different techniques. In this study, we applied VBM methods and identified in HSP patients at baseline a significant volume reduction in the right pre-frontal cortex and bilaterally in the thalamus in HSP patients at baseline, while no alteration was detected in the GM of the primary motor areas. This finding supports the idea that the main target of HSP pathological mechanism is not directly the primary motor cortex as other advanced MRI and neuropathological studies already reported (Kassubek et al., 2006; Denora et al., 2016; Parodi et al., 2018b; Coarelli et al., 2019; Graça et al., 2019).

The variations that we observed in the pre-frontal cortex and in the thalami from a pathophysiological perspective can be considered as involvement of associative motor areas, but it is well known that the same areas are also involved in the connections of the wide extra-motor world. Okubo and colleagues reported a SPECT study in HSP and demonstrated both at cross-sectional and at longitudinal analysis a decreased perfusion in various cerebral areas. Specifically, they found decreased perfusion in the lateral frontal cortex and in the thalamus at long-term follow-up that correlated with clinical cognitive deterioration (Okubo et al., 2000). The investigation of possible correlation between thalami reduction and cognitive involvement in HSP was subsequently pursued also with advanced MRI techniques (França et al., 2007; França et al., 2012; Faber et al., 2018). It has been frequently stressed that the deterioration of cognitive functions is not exclusive of complicated HSPs but can be observed also in the so-called pure forms (Balconi and Bortolotti, 2013; Agosta et al., 2015; Martinuzzi et al., 2016; Menke et al., 2018; Jacinto-Scudeiro et al., 2019). Systematic studies investigating cognitive performances in SPG4 reported dysfunctions in 41 to 96% of HSP patients, depending on the test and the domain investigated (Rezende et al., 2015), and an abnormal fMRI connectivity between the middle frontal and the orbitofrontal gyri (Liao et al., 2018).

In this study, we did not detect significant longitudinal changes by VBM but we cannot exclude that such negative result is due to limits intrinsic to the methodology and to the small size of our cohort. For the latter reason, we are planning to expand our study to a larger HSP group and to apply more detailed methods of GM analysis, such as the cortical thickness analysis, similar to previous studies in ALS (Kwan et al., 2012) and to a recent one on HSP (Faber et al., 2018) showing interesting preliminary data.

Our MRS data confirm that HSP is a disease not primarily affecting the neurons of the primary motor regions. NA-acetylcholine is synthesized from choline and can be reduced in many neurological diseases, including neurodegenerative conditions such as Alzheimer’s disease, as it is an index of concentration and/or functionality of neurons. The NAA/Cr ratio in our study is reduced in the pre-frontal areas, but not in the pre-central motor regions. This finding is confirmed also by comparing the genetically homogeneous group of SPG4 patients to normal subjects. At baseline, the only MRS variation involving the pre-central regions is an increase of mI/Cr ratio observed in patients. The same finding is the only difference identified at baseline in non-SPG4 patients compared to SPG4. Myo-inositol is considered as an index of membrane activity, referred not only to neurons/axons but also to oligodendrocytes, expressing possibly reparative attempts. Even if our group of patients includes various SPG types, the statistical variation of mI/Cr at baseline probably expresses a slow evolution of the HSP pathology.

Other MRS studies reported a stable level of Cho in HSP when compared to the normal subjects or eventually a reduction, reflecting an impairment of membrane turnover and/or prevailing cell loss. On the contrary, our study describes an increase of Cho/Cr ratio in patients compared to HC in the pre-central region in absence of an evident reactive/inflammatory brain activity that usually is expressed in MRS as increase of Cho. Our MRS data reflect the extreme inhomogeneity of the reports in literature on HSP. The heterogeneity in terms of SPG forms considered, localization of the sampled regions, the influence of the clinical extramotor involvement, in particular in the cognitive dominion, are the main limits in studies with MRS. Only Erichsen and colleagues analyzed a homogeneous group of SPG (eight SPG4 patients), placing the MRS boxes of sampling in areas similar to ours (left frontal white matter and pre-central gyrus). The results are at variance with ours: they found a reduction of the Cho/Cr in the pre-central cortex, in contrast to our study; decreased NAA/Cr in the frontal regions, similar to our study, but also in the motor cortex, where we did not find any change; and increased mI/Cr in patients, but without statistical significance, while our increased mI/Cr values are statistically significant. The conclusion is that the complexity of these results must be considered with caution given the many potentially confounding variables, the relatively small size of the observed changes, the variability also seen in the controls, and the heterogeneity and small number of patients studied. To overcome at least some of these problems, cooperative multicentric studies pooling data from various centers are recommendable once the technical aspects of the procedure are agreed upon and harmonized. A possible solution, which however entails a very expensive acquisition time, could be the application of techniques of absolute MRS quantification, as reported over 10 years ago in a single report on only one HSP patient (Dreha-Kulaczewski et al., 2006).

From the complexity and heterogeneity of our VBM and MRS data, the constantly emerging element is the apparent absence of a direct involvement of the primary motor cortex in HSP patients, in agreement with similar neuroimaging and neuropathologic reports. Conversely, our DTI data indicate an intense alteration of brain WM that includes also the primary motor cortex. As reported in literature, DTI indexes are characterized by decreased FA and quite anatomically corresponding increased MD, with a variable but often corresponding increase of RD and AD that is to be globally considered as an expression of WM reduction in fiber density and homogeneity in spatial orientation, with myelin degradation over axonal degeneration (França et al., 2012; Garaci et al., 2014; Schneider-Gold et al., 2017).

Our VBM and MRS data at baseline highlight another element: a possible widespread brain involvement in HSP patients not focused on the primary motor areas. This is strongly confirmed by DTI. These results lead to include HSP among the non-regional or neural system specific pathologies; that is intriguing, but not surprising given what has been shown in other neurodegenerative pictures, such as in Parkinson’s and Alzheimer’s disease and in ALS (Senda et al., 2011; Agosta et al., 2012; Menke et al., 2018).

The paradigm of FA reduction and MD increase is usually correlated to a reduced axonal density, referred to as a decrease in the number of fibers and/or in the thickness of their myelin sheaths, that causes a disarrangement in the spatial orientation of the fibers with an increase of the extracellular spaces (Werring et al., 1999; Rosas et al., 2006), as sustained by findings from a comparative study between DTI and gross pathological features (Mottershead et al., 2003). The novel data from our study are the significant time-related changes moving on longitudinal analysis of DTI indices in the opposite direction to what we would expect from a progressing disease: FA increased and MD decreased in widespread areas. Such DTI changes were never reported before; rather, some authors underlined the absence of regions with significant FA increase in HSP (França et al., 2012).

We should take into account the small number of patients completing the follow-up, but we should also consider that the comparison with the controls sampled with the same methodology and after the same time interval strengthens the significance of the results. Interference by technical problems is avoided by this approach, given the data stability seen in our cohort of HC in whom no DTI variation was detected. The WM DTI changes in our patients involved scattered cerebral regions, such as the superior longitudinal fasciculus and pre-central and post-central WM, and they could be considered as improvement in terms of functionality. This is supported by the decrease of RD, while AD showed an alternating time effect.

Understanding the pathophysiological mechanisms of these DTI variations is not easy and requires further targeted investigations. On the basis of the classical interpretations of DTI indexes, we could speculate that we observed an increase of homogeneity of WM fibers and a reduction of free spaces between the fibers themselves, that could represent a reorganization attempt of the residual fibers at the microscopic level. These findings have no clinical correlation as we didn’t observe any longitudinal variation of SPRS data.

While studies with advanced MRI on longitudinal progression are common in other neurodegenerative diseases, such as ALS (Agosta et al., 2009a, b; de Albuquerque et al., 2017; Menke et al., 2018), they are rare in HSP (Garaci et al., 2014) or conducted in very small sample sizes: Dreha-Kulaczewski and colleagues (Dreha-Kulaczewski et al., 2006) described a single patient with HSP-TCC studied with MRS and DTI with a 5-year follow-up, showing variations in the frontal WM, thalamus, and pyramidal motor system. Most of the studies that recruited a larger patient cohort was designed to implement only one or two MRI techniques (França et al., 2007) or presented intergroup comparison with controls scanned only at baseline or only at follow-up. To date we are not aware of any other study that scanned patients and controls in parallel over time.

None of the metrics reported in previous studies based on DTI or on WM volumes displays any correlation with disease duration in HSP (Faber et al., 2018), suggesting that the clinical indicators are either moving at a different pace or are not sensitive enough to detect the finer variations that DTI can identify (Fink, 2013; Faber et al., 2018). Clinical scales, albeit useful and validated, often display coarse sensibility and possibly ceiling/floor effects. HSPs are very slowly and not uniformly progressive disorders and this, as their rarity, greatly limits the ability to conduct randomized controlled trials in a reasonable time frame (6–24 months). In a cohort of 116 HSP patients, the analysis of the progression revealed in the subjects with slower course a less severe outcome, which was not explained by age at onset or genotypes (Parodi et al., 2018b). In another study on SPG7 patients, a rapid progression was detected in the first stage of the disease, after which the patients reached a plateau (Coarelli et al., 2019). Other authors reported a progression of symptoms during follow-up only in 58.3% cases, while 41.7% showed no motor worsening (Ortega and Rosemberg, 2019). Disease-ameliorating processes promoting the recovery of damaged axons or functional compensation through neuroplasticity could contribute to slow down the rate of neurodegeneration. A neuropathological study on HSP patients can be illuminating in the interpretation of our DTI data: Parodi and colleagues (Parodi et al., 2018b) described the high density of very small fibers with thin myelin in the brain and the spinal cord of HSP patients, interpreted as evidence of regeneration attempts within the CST. In this respect, the potential role of advanced MRI as a biomarker for HSP is still to be supported (Graça et al., 2019).

At the longitudinal analysis we found no correlation between our DTI data and treatment history (physiotherapy, chemodenervation, and anti-spasticity medications) unlike what is highlighted in other reports on chemodenervation with botulin toxin, in which improvement of DTI indexes correlated with clinical conditions (Xi et al., 2018).

Prognostic data for individual SPG types are also scarce and do not help to clarify our DTI data as the median disease duration until loss of independent walking is estimated in 22 years (Shribman et al., 2019), leading to consider our follow-up interval (about 30 months) as too short to be able to evaluate consistent variations in clinical and para-clinical parameters. Still our results allow us to confirm that the DTI technique might offer a more sensitive way to monitor and document changes over time.

Some studies report an atypical increase of FA values in the absence of appreciable clinical improvement in neurodegenerative diseases, such as Huntington’s disease (Douaud et al., 2009) and in chronic secondary neurodegenerative evolution related to Wallerian degeneration (Pierpaoli et al., 2001). The interpretation offered in these reports is the possible loss of crossing fibers that can induce an apparent prevalence of co-oriented fibers, technically leading to an increase in FA. These reports suggested the application of modern DTI analysis of WM, such as fixel-based techniques, disentangling crossing from non-crossing fibers (Raffelt et al., 2017), or deconvolution model (Dell’Acqua and Tournier, 2019). With these modern techniques, it is possible to quantify fiber density and fiber bundle cross-section in order to obtain a more complete WM structural morphometry, combining information from both within-voxel microscopic fiber density and macroscopic fiber bundle cross-section (Raffelt et al., 2017; Genc et al., 2018). These techniques are really promising, but the FA increase in the longitudinal analysis that we observed displays a fundamental difference with respect to a possible effect due to the reduced crossing fibers: while in the reports on Huntington’s disease and Wallerian degeneration the increased FA corresponded to an increase of the MD values, that express the enlargement of the inter-axonal spaces due to degenerative processes, in our study, the increased FA is associated to a parallel decrease of MD. For this, it is possible to sustain that our results reveal a quite different scenario.

Our study presents several limits, the main of which are the mixed types of SPG forms and the low number of patients, even considering the rarity of the disease and the difficulty in recruiting patients. The variability in clinical presentation, onset of the symptoms, and progression of the disease would be taken into account when analyzing MRI data, that need to extend the number of participants of each single type of genetical and phenotypical form. This could be relevant in particular for MRS and VBM analysis that hardly depend on the sampled population.

Another limit is the analysis of our DTI data themselves: on longitudinal analysis, the whole-based voxelwise paired TBSS analysis did not show significant differences in the patients’ DTI indexes, while our statistical results are based on anatomic regional selection. Whole-based voxelwise paired TBSS analysis has high spatial resolution, but probably the small size of our sample gave insufficient statistical power. Voxelwise analysis itself is not exempt from limitations, depending on the possible residual misregistration when warping the diffusion images into a common standard space: the method described in this paper depends heavily on having reliable normalization in patients who can present diffuse or sectional brain atrophy that can influence the final results. The voxel-by-voxel comparison is only possible if voxels can be assumed to represent exactly the same anatomic region in every subject and the normalization process does not introduce or remove bugs (Schwartzman et al., 2005). For these reasons, we added to the analysis a ROI approach that allowed us to obtain specific DTI values from limited brain districts with high sensitivity. But, this approach too is an averaging measure of multiple voxels, so we cannot exclude a partial volume averaging effect in the application of a general atlas that uses automated templates. The most important risk is to include structures that are a mixture of gray and white matter. Therefore, future studies should include single portions of bundles or anatomic district divided in sub-segments, free from averaging volume effect, as previously manually attempted (Martinuzzi et al., 2016).

Our results require confirmation on larger and genetically homogeneous cohorts; therefore, we agree with the proposal of Parodi and colleagues on the importance of creating networking study groups to focus on the neuroimaging approach to HSP (Parodi et al., 2018b). This could help in overcoming genetic, clinical, and technical limits and in identifying sensible and specific biomarkers, needed to proceed with therapeutic trials.

## Data Availability Statement

The datasets generated for this study are available on request to the corresponding author.

## Ethics Statement

The studies involving human participants were reviewed and approved by Institutional Ethics Committee of the IRCCS “Eugenio Medea” Research Institute (#63/09CE). Written informed consent to participate in this study was provided by the participants’ legal guardian/next of kin.

## Author Contributions

DM, MV, and AM: design and plan of the study, collection of the clinical data, discussion, interpretation, and description of the results. FF and GA: image analysis of the advanced MRI and interpretation of the data, discussion, interpretation, and description of the results. CS, GG, and GPa: collection of the clinical data. AB and GPi: acquisition of the MRI exams. AC: statistical analysis of the data. SD, PT, and FL: critical revision of the results and discussion. The manuscript was written and edited by all the authors.

## Conflict of Interest

The authors declare that the research was conducted in the absence of any commercial or financial relationships that could be construed as a potential conflict of interest.

## Footnotes

1 http://www.fmrib.ox.ac.uk/fsl
